# Identifying priority reserves favors the sustainable development of wild ungulates and the construction of Sanjiangyuan National Park

**DOI:** 10.1002/ece3.9464

**Published:** 2022-11-03

**Authors:** Feng Jiang, Jingjie Zhang, Pengfei Song, Wen Qin, Haijing Wang, Zhenyuan Cai, Hongmei Gao, Daoxin Liu, Bin Li, Tongzuo Zhang

**Affiliations:** ^1^ Key Laboratory of Adaptation and Evolution of Plateau Biota Northwest Institute of Plateau Biology, Chinese Academy of Sciences Xining Qinghai China; ^2^ University of Chinese Academy of Sciences Beijing China; ^3^ Qinghai Provincial Key Laboratory of Animal Ecological Genomics Xining Qinghai China; ^4^ State Key Laboratory of Plateau Ecology and Agriculture Qinghai University Xining Qinghai China

**Keywords:** conservation strategies, core reserve area, large wild herbivores, maximum entropy model, Sanjiangyuan national park

## Abstract

Sanjiangyuan National Park (SNP), the first national park in China, is one of the most important biodiversity conservation areas in the Sanjiangyuan National Nature Reserve (SNNR) and even the world. The threatened ungulates play an irreplaceable role in maintaining the ecosystem diversity and stability in SNNR. Here, based on 1434 occurrence records of six ungulates, the maximum entropy model, with two different strategies, was utilized to determine the priority reserves. The results indicated that the priority reserves in SNNR was mainly located in and around SNP, which were mainly distributed in the middle east, middle west, and southwest of SNNR. Six ungulates shared preference for altitude ranging 4000–5000 m, the average annual temperature below −3.0°C, and average annual precipitation ranging 200–400 mm on meadow, steppe, and unused land. The proportion of high and medium suitable areas for ungulates in SNP was higher than that in SNNR. As the SNP is not contiguously spaced in space, and some core wildlife habitats are not included, it is suggested to optimize the functional areas and adjust the boundary range on the basis of the pilot scope of SNP, so as to enhance the integrity and connectivity of each functional area.

## Graphical abstract

Two strategies were used to analyze the overlap of suitable habitats.Core suitable areas of herbivores were similar in SNP and SNNR based on both strategies.SNP is the most suitable distribution area for six herbivores in SNNR.

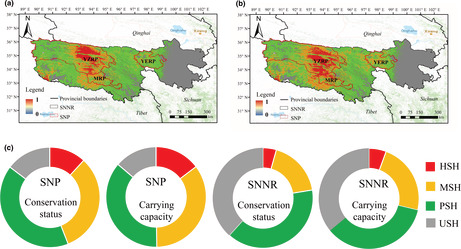

## 1 INTRODUCTION

Sanjiangyuan National Park (SNP), the China's first national park, is an important area of biodiversity with global significance, and its natural ecosystem is typical and representative of the Qinghai‐Tibet Plateau (Han et al., 2019). SNP maintains good authenticity of wildlife and the integrity of species habitat, and large wild ungulates have a high value of biodiversity and ecosystem stability. In recent years, with the establishment and protection of multi‐type nature reserves such as national parks and reserve, the number of wild ungulates has been increasing steadily, while the endangered status of some ungulates has been declining (Zhao et al., 2020). However, the conflict between wild ungulates and livestock is increasing, and the influence of herbivores on the grassland ecosystem become more and more prominent. Meanwhile, the large wild ungulates have to face harsh living conditions of the Qinghai‐Tibet plateau, so they are still exposed to more environmental stress. Identifying priority reserves of large wild herbivores considering both protection level and carrying capacity of grassland is the first step of animal conservation and also benefits to the balance and stability of the entire ecosystem in SNP.

Here, the Tibetan antelope (*Pantholops hodgsonii*), the white‐lipped deer (*Cervus albirostris*), the wild yak (*Bos mutus*), the Tibetan gazelle (*Procapra picticaudata*), the Tibetan wild ass (*Equus kiang*), and the Bharal (*Pseudois nayaur*) are the six large wild ungulates with the largest distribution and relatively largest population in SNP (Zhang et al., 2022). These species represent the most extensive and typical species of large herbivores in the SNP, and they depend on the plateau grassland ecosystem for nutrition and are part of the carrying capacity of the plateau grassland (Fox et al., 2009). Our preliminary estimate of the population of these six large wild herbivores is about 187,000 in the SNP, and they are the main populations of large wild herbivores in Sanjiangyuan National Park (Zhang et al., 2022). In addition to the Bharal, the other five ungulates are endemic to the Qinghai‐Tibet Plateau (Jiang et al., 2016; Signore & Storz, 2020). The Tibetan antelope is the flagship species on the Qinghai‐Tibet Plateau. From the 1950s to the 1990s, illegal poaching led to a 90% drop in the Tibetan antelope population (Leclerc et al., 2015). Since the 1990s, due to a series of conservation studies from international organizations and the strictest protection measures implemented from China, the population of this species has recovered greatly (Du et al., 2016; Zhao et al., 2020), although not to historical population levels. The Tibetan gazelle is a key large wild herbivore in the SNP (Jiang et al., 2019), while it is more widely distributed and abundant than the Tibetan antelope. Both species play an important role in maintaining the stability of the regional alpine steppe and alpine meadow ecosystem. The Tibetan wild ass is the only odd‐toed ungulate living on the Qinghai‐Tibet Plateau and is the largest of all the wild asses in the world. More than 90% of the global Tibetan wild ass population are found in China, including most of Qinghai (Shah et al., 2015). The wild yak, one of the largest bovine animals, historically had a wide distribution, occupying the north slope of the Himalayas, Kunlun Mountains, and their adjacent mountains. In the past 100 years, the habitat area and population of wild yaks have declined sharply due to human poaching and habitat destruction (Schaller & Liu, 1996). At present, this species is isolated and dispersed in the Qinghai‐Tibet Plateau (Liang et al., 2017). The white‐lipped deer is a typical large deer on the plateau. Human poaching and habitat fragmentation have also led to a sharp decline in the population and an island‐like distribution. Compared with other wild ungulates in SNP, the Tibetan wild ass and the wild yak have the largest body size and large food intake, hence have the most vigorous demands for grassland resources. Similar to the wild yak and Bharal, the white‐lipped deer had a relatively narrow range and is highly selective to habitat.

In addition, as a sensitive and ecologically fragile region of global climate change, the Qinghai‐Tibet Plateau amplifies the climatic fluctuations caused by climate change, which in turn makes ungulates in the region very sensitive to climate change (Pei et al., 2019; Wu et al., 2017). The study of the resource and habitat partitioning of six large herbivores in the SNP, as well as the carrying capacity of grassland for large herbivores and the establishment of early warning mechanism could be of great help to the development of wildlife and habitat protection and management countermeasures. These could result in effective protection is as an important way to promote the health and stability of grassland ecosystem. A suitable living environment can provide a space for wildlife to survive, reproduce, and take refuge, and the research on the spatial distribution of wildlife is the basis of species protection (Gong et al., 2020). Therefore, it is necessary to understand the distribution, abundance, and spatial and temporal dynamics of rare wild herbivores in the park, the interaction between species, and the dynamic changes of the suitable habitat for species. With the popularization of geoinformatics and multivariate analysis statistics, the maximum entropy (MaxEnt) model has been proved to be a model with better prediction results by various studies (Hernandez et al., 2006). This model is a density estimation and species distribution prediction model based on the maximum entropy theory and combines the actual geographic distribution information of the target species with constraints such as environmental variables in the study area (Phillips et al., 2006). The habitat suitability evaluation model can be used to evaluate the habitat suitability of specified species under given environmental conditions and predict the potential habitat of species (Phillips & Dudik, 2008). Combining historical and future geographic and climatic data, it is also possible to predict the migration paths and future potential distribution status for protected species (Jiang et al., 2020).

In this paper, the MaxEnt model was used to evaluate the habitat suitability of six dominant ungulates in the Sanjiangyuan area. The objectives of this study were to: (i) identify ecological determinants of habitat suitability for every species; (ii) assess the priority conservation areas for every species and evaluate how different conservation strategies would affect the optimal choice of suitable core conservation areas; (iii) identify core protection areas in the Sanjiangyuan region and suitable distribution areas outside SNP. The results of the study will benefit the conservation of ungulate species and provide empirically robust support for the selection and protection of potential wildlife habitats in the Sanjiangyuan area, as well as the planning and construction of China's first national park.

## 2 MATERIALS AND METHODS

### 2.1 Study area

The SNP located in the hinterland of the Qinghai‐Tibet Plateau, which is known as the “Water tower of Asia” of the world (Han et al., 2019). The construction of the SNP is not only a model for the construction of high‐altitude national parks in the world but also the key to the conservation of plateau species diversity. The SNP has an area of about 123,100 km^2^, accounting for 31.2% of the SNNR, involving four counties of Zadoi, Qumarleb, Madoi, and Zadoi and the area under the jurisdiction of Hoh Xil Nature Reserve, with 12 towns and 53 administrative villages (Jiang et al., 2019). It preserves the better authenticity of wildlife and the integrity of species habitat with a variety of plateau endemic animal populations.

The Park has a variety of ecological systems, such as glaciers, snow‐capped mountains, high‐altitude lakes and wetlands, and alpine grasslands and meadows. Different ecological types breed rich and unique plateau wildlife resources, which is known as the “Alpine biological germplasm resource bank”. Wildlife is an important part of the plateau ecosystem and plays an irreplaceable key role in maintaining the diversity and stability of the whole ecosystem. Among them, large wild herbivores as primary consumers, they play an irreplaceable key role in maintaining the diversity and stability of the entire ecosystem.

### 2.2 Occurrence records collection and processing

In recent years, Ecological Niche models (ENMs) have been used to simulate the habitat suitability and potential suitable spatial distribution of target species with geographical environmental factors (Phillips et al., 2006; Phillips & Dudik, 2008). At present, the MaxEnt model, a type of ENMs, is widely used due to simple operation and good simulation results (Fernandez et al., 2015; Wauchope et al., 2017). The model requires two sets of data: the spatial distribution site of the target species and the environmental variables of the study area. The prediction results of the model without parameter optimization may have serious fitting deviation, which is not conducive to the simulation of the potential suitable habitat of species (Guevara et al., 2017; Polce et al., 2014). Therefore, occurrence records and environmental variables were screened, and the parameters of the model were also optimized before the model simulated the suitable spatial distribution of six large wild herbivores in this study.

From 2016 to 2018, we conducted an extensive background survey of wildlife in the Sanjiangyuan area through the point and line transect methods with GPS locator (Garmin, China), telescope (Olympus, Japan), telephoto camera (Sony, Japan), and unmanned aerial vehicle (DJI, China). We set up survey lines randomly along roads and valley and maximized the coverage of different habitat types using ArcGIS software (v10.2). The wildlife names and their numbers, the distance to the observed species, geographical locations (latitude and longitude), habitat types, image data, and threat factors of the wildlife were recorded in detail.

**FIGURE 1:**
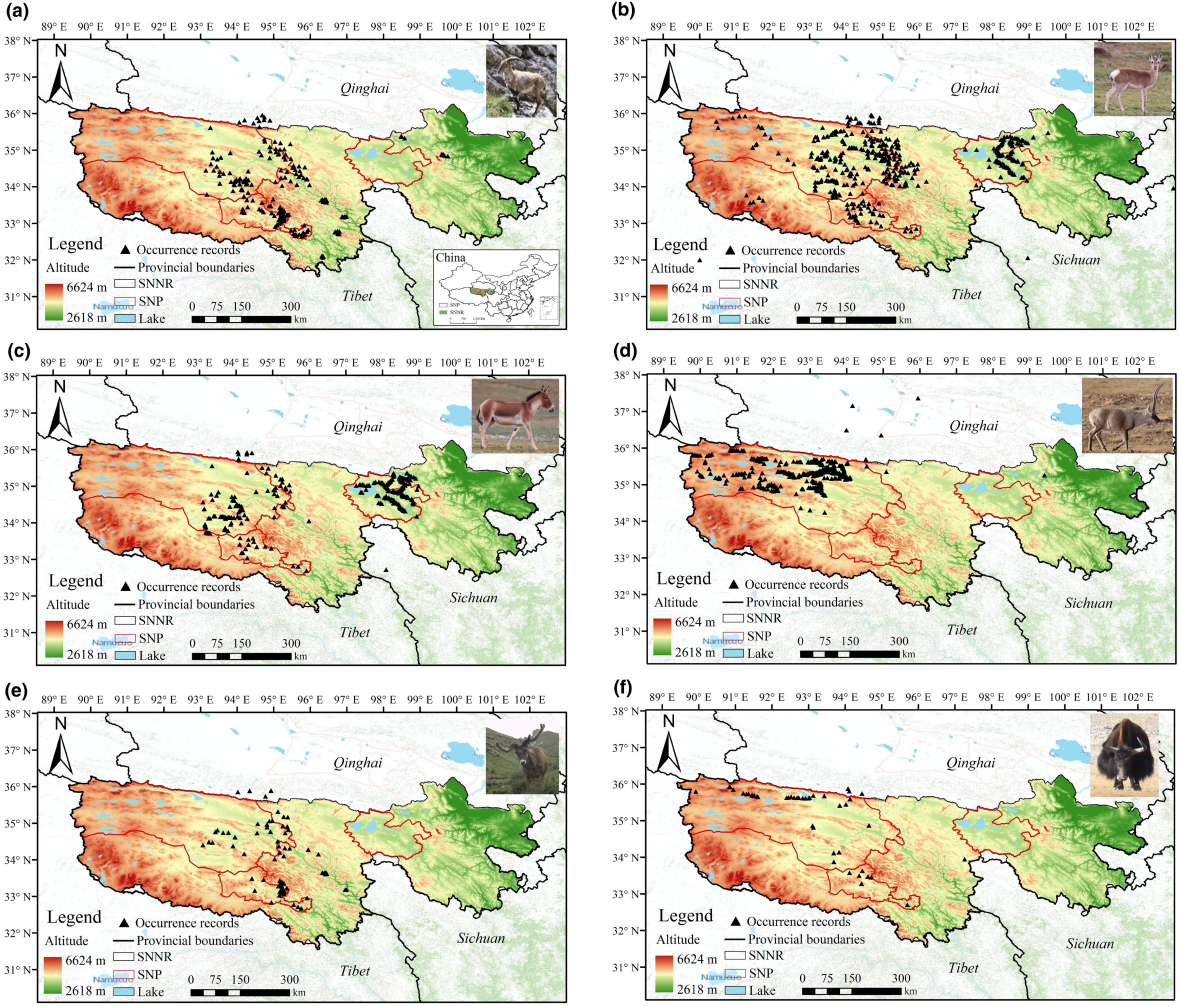
Occurrence records of wild herbivores in and around Sanjiangyuan National Nature Reserve. (a) Bharal; (b) Tibetan gazelle; (c) Tibetan wild ass; (d) Tibetan antelope; (e) white‐lipped deer; (f) wild yak.

To reduce the negative impact of spatial autocorrelation between occurrence records on the establishment of niche models, and to improve the reliability of simulation results, the study used resolution distance to screen the distribution points of six large wild herbivores (Chamaillé et al., 2010; Polce et al., 2014). The ArcGIS (v10.2) was used to set the resolution distance as 1 km, and the distribution points that appeared close or repeated in the same grid were deleted. After screening, 205 occurrence records of the Bharal, 400 of the Tibetan gazelle, 323 of the Tibetan wild ass, 386 of the Tibetan antelope, 70 of the white‐lipped deer and 49 of the wild yak were remained, respectively, for the niche model evaluation in the study (Figure 1). The occurrence records were input into the table in the format of “species‐longitude‐latitude” and was saved as “.csv”.

### 2.3 Collection and filtering of environment variables

A total of 77 environment variables closely related to six large wild herbivores were collected for species distribution models, which were divided into four categories (Table S1). The autocorrelations and multiple linear duplications among environment variables might affect the prediction results of the model (Carlos‐Júnior et al., 2015). To reduce the overlap of information between variables, SPSS v22 software was used to calculate the environmental attribute values of six herbivores and calculate the correlation coefficient to screen the environmental variables. The variables with high correlation (|*r*| ≥ 0.80) were eliminated, and those with low correlation and more biological implications were introduced into the model operation (Johnson et al., 2016; Kumar et al., 2015), so as to improve the accuracy of the simulation results of the niche model. SPSS v22 was used for all statistical analysis of environmental variables.

### 2.4 Parameter optimization and ecological niche modeling

The MaxEnt v3.3.3 k model was used in this study. To obtain the optimal parameters of the species distribution modeling for six large wild herbivores in the Sanjiangyuan area, so as obtain a more accurate prediction result, this study set and screened different combinations of feature class selection (FC) and regularization multiplier (RM) parameters, which were the two main parameters and influenced the predictive performance of the MaxEnt model (Guevara et al., 2017). The FC included linear, quadratic, hinge, product, and threshold features, which were regularly combined based on the number of sample points. The RM value was set between 0.5 and 5 with 0.5 intervals. ENMTools User Manual v1.4.3 was used to calculate AICc and BIC scores under different parameters. Combined with the smoothness of response curves, the parameters of AICc and BIC with the lowest scores and relatively smooth response curves were taken as the optimal parameters for constructing species distribution modeling of six large wild herbivores.

The data of species distribution points were randomly divided into two groups. One set was 75% as the training set for the model construction, and another set of 25% records was used as test sets for model validation (Tanner et al., 2017). The average probability from 10 model runs were regarded as the final predicted value (Jiang, 2018; Polce et al., 2014). Meanwhile, receiver operating characteristic (ROC) curves and AUC value (area under ROC curve) were used to evaluate the accuracy of MaxEnt model. A larger AUC value, ranging from 0 to 1 indicates higher reliability and accuracy of the model.

### 2.5 Analysis of core and overlap areas of large wild herbivores

The habitat probability distribution layer of six large wild herbivores generated by MaxEnt simulation was imported into ArcGIS (v10.2), and the existence probability value ranged from 0 to 1. The layer data was divided into four levels by using reclassification function of ArcGIS. The existence probability of ≥0.6, 0.4–0.6, 0.2–0.4, and <0.2 were respectively indicated as high suitable, medium suitable, low suitable, and unsuitable areas (Convertino et al., 2014; Padalia et al., 2015). The high and medium suitable areas were taken as the priority conservation areas for large wild herbivores (Jiang et al., 2019).

Each of the six species was given weight by two different strategies: the protection level of the six large wild herbivores (Table 1) and the carrying capacity (namely the sheep unit in the meadow capacity) (Table 2). The suitable distribution of the six herbivores were overlaid with different weighted values. The protection level referred to five classification methods (Table 1). The calculation formula was as follows:

$$x=\frac{\sum_{i=1}^{n}c_{i}x_{i}}{\sum_{i=1}^{n}c_{i}}$$

Here, *x* represents the synthetic probability of existence. *x*_*i*_ represents the existence probability value of the distribution layer of each species. *c*_*i*_ represents the final weight value of each species.

**TABLE 1:** The six species were given weight by the protection level of the six large wild herbivores

Species	LEPS^a^		China red list^b^		Endemism^c^		IUCN red list^d^		CITES^e^		Weighted values^f^
	Class	Value	Status	Value	Class	Value	Status	Value	Status	Value	
Bharal	II	0.7	LC	0	No	0	LC (2014)	0	III	0.3	0.20
Tibetan gazelle	II	0.7	NT	0.25	Yes	1	NT (2016)	0.25	Null	0	0.44
Tibetan wild ass	I	1	NT	0.25	Yes	1	LC (2015)	0	II	0.7	0.59
Tibetan antelope	I	1	NT	0.25	Yes	1	NT (2016)	0.25	I	1	0.70
white‐lipped deer	I	1	EN	0.75	Yes	1	VU (2014)	0.5	Null	0	0.65
Wild yak	I	1	VU	0.5	Yes	1	VU (2014)	0.5	I	1	0.80

^a^ LEPS means the List of endangered and protected species of China. *The Animal protection law of the People's Republic of China* (adopted in 1988 and revised in 2018) divided wildlife into first‐class (I) and second‐class (II) national protected animals.

^b^ China Red List referred to the Red List of China's Vertebrates (Jiang et al., 2016).

^c^ IUCN Red List referred to IUCN Red List Categories and Criteria (https://www.iucnredlist.org/). The date of last assessment for species is different.

^d^ Protected species is included in one of three lists in the Convention on International Trade in Endangered Species of Wild Fauna and Flora (CITES), called Appendix I, Appendix II and Appendix III, which was valid from 2017.

^e^ Endemic species were mainly considered to be endemic to the Qinghai‐Tibet Plateau.

^f^ The final weighted value was the average value of five protection levels.

**TABLE 2:** The six species were given weight by the sheep unit in the meadow capacity

Species	Sheep unit^a^
Bharal	1.0
Tibetan gazelle	0.5
Tibetan wild ass	4.0
Tibetan antelope	1.0
White‐lipped deer	3.0
Wild yak	5.0

^a^ The Conversion standard of sheep unit for six large wild herbivores referred to Li et al. (2019).

## 3 RESULT

### 3.1 Environmental variable screening and model accuracy analysis

The filtered environment variables with small correlation were used for the MaxEnt model operation (Table S2). After selecting the optimal parameter (Table S3), according to the ROC curve generated by the MaxEnt model (Figure S1), the average AUC_test_ values of the six herbivores with 10 repeated runs were all over or close to 0.9. The standard deviations were all lower than or close to 0.05 (Figure S1, Table S3). This indicated that the spatial suitability distribution prediction results were very good and had high accuracy.

### 3.2 Analysis of typical ecologically determined environmental variables of different species

**FIGURE 2:**
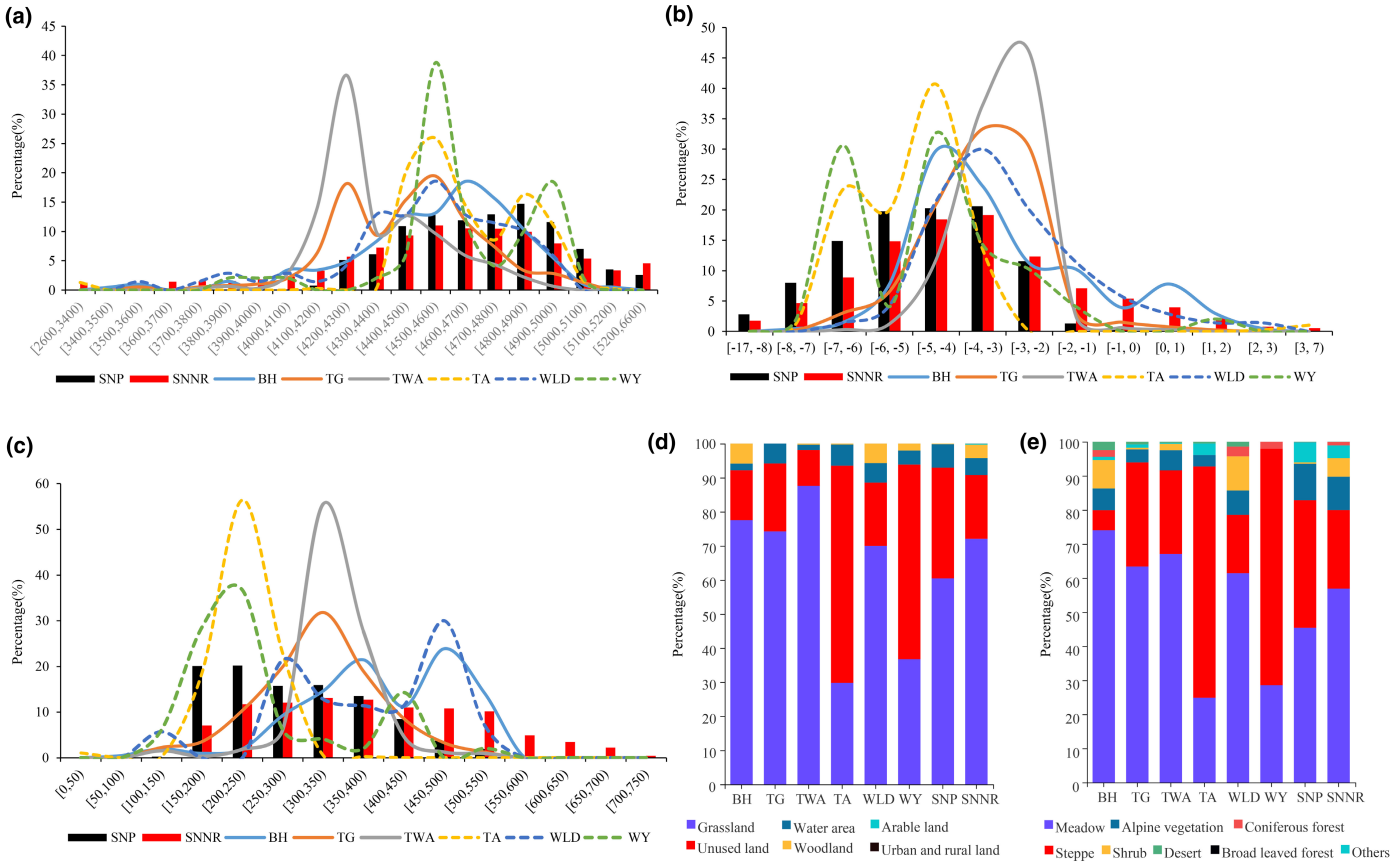
Analysis of environment variables for six wild herbivores in Sanjiangyuan area, including altitude (a), average mean temperature (b), annual precipitation (c), land use types (d), and vegetation types (e). BH, TG, TWA, TA, WLD, and WY stand for the Bharal, the Tibetan gazelle, the Tibetan wild ass, the Tibetan antelope, the white‐lipped deer, and wild yak, respectively.

The Bharal, the Tibetan gazelle, the Tibetan wild ass, the Tibetan antelope, the white‐lipped deer, and the wild yak had the highest proportion at altitude of 4400–4900 m, 4200–4700 m, 4100–4600 m, 4400–5000 m, 4300–4900 m, and 4500–5000 m, respectively (based on a single interval of 10% and a cumulative proportion higher than 70%) (Figure 2a; Table S4). Among them, the Bharal, the Tibetan antelope, and the white‐lipped deer had higher similarity with SNP and SNNR in terms of altitude (*r* ≥ 0.8, *P* < 0.01) (Table S5).

The above six species had the highest proportion in the average mean temperature of [−5, −1), [−5, −2), [−5, −2), [−7, −3), [−5, −1), and [−7, −2) °C, respectively (Figure 2b; Table S4). Among them, the Bharal, the Tibetan gazelle, and the white‐lipped deer had higher similarity with the SNP and the SNNR (Table S6). In addition, those species had the highest proportion in annual precipitation of 300–550, 200–400, 300–400, 150–300, 250–500, and 150–250 mm, respectively (Figure 2c; Table S4). Although Tibetan gazelle have the closest distance in the distance matrix with the SNP and the SNNR, their correlation coefficients were all less than 0.8 (Table S7).

In terms of land use types, the Bharal, the Tibetan gazelle, and the Tibetan wild ass had the highest proportion of distribution sites on grassland, which were 77.6%, 74.2%, and 87.6%, respectively (Figure 2d). While, the Tibetan antelope, the white‐lipped deer, and the wild yak had the highest proportion of distribution points on grassland and unused land, with 93.5%, 88.6%,and 93.9% accumulative percentage, respectively. All of them had high similarity with the SNP and the SNNR (Table S8).

Among the vegetation types, the Bharal had the highest proportion of distribution sites on meadow. While, the other five herbivores had the highest proportion on grassland and meadow (Figure 2e). The Tibetan gazelle and Tibetan wild ass had high similarity with the SNP and the SNNR. The Bharal and the white‐lipped deer have a high similarity with the SNNR (Figure 2e; Table S9).

### 3.3 Assessment of potential suitable geographical areas and core area of wild herbivores

**FIGURE 3:**
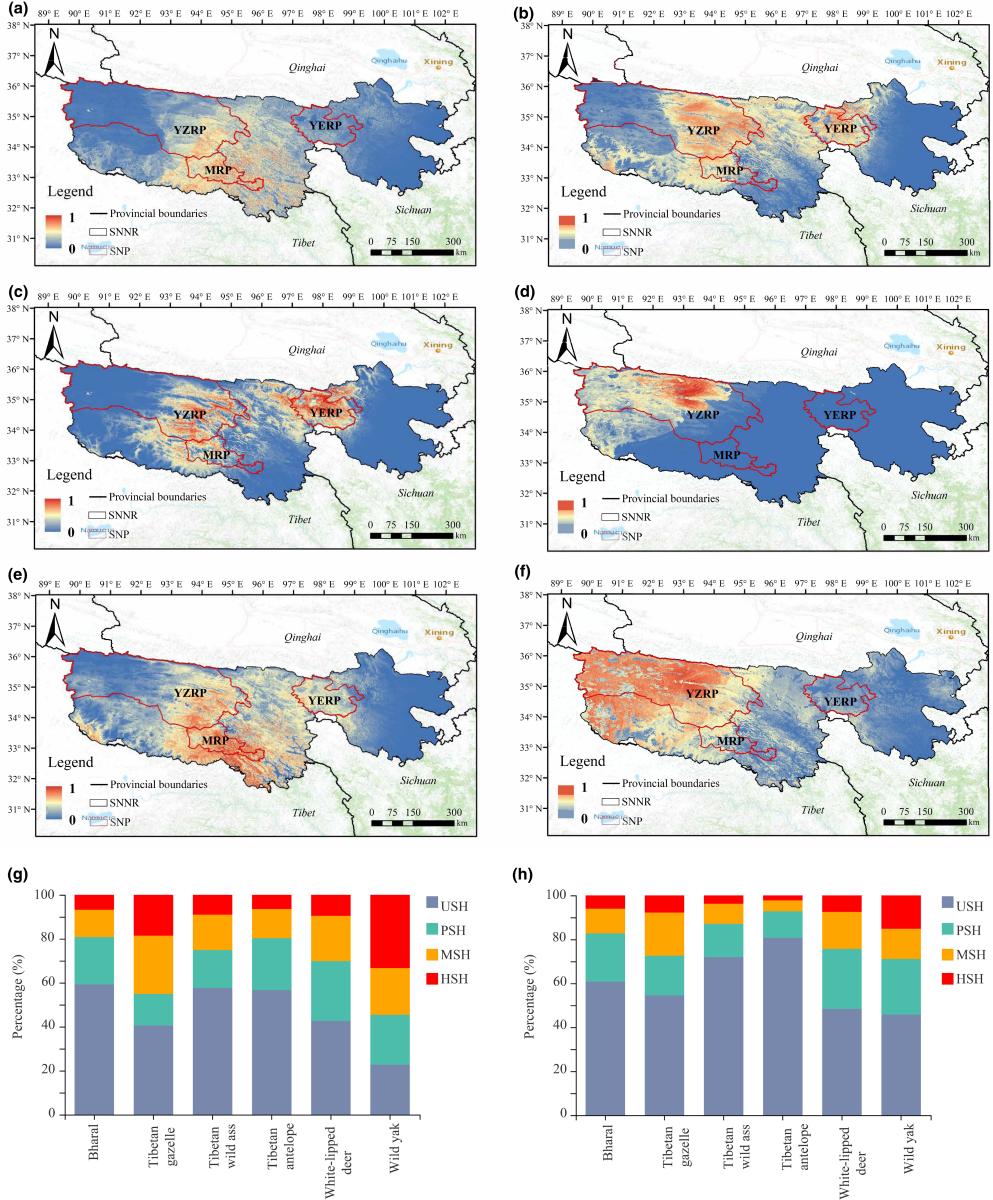
Suitable habitat for the six wild herbivores in the Sanjiangyuan area. (a) Bharal; (b) Tibetan gazelle; (c) Tibetan wild ass; (d) Tibetan antelope; (e) white‐lipped deer; (f) wild yak. Habitat composition of the six wild herbivores in SNP (g) and SNNR (h). YZRP, MRP, and YERP stand for Yangtze River park, Mekong River park, and Yellow River Park, respectively.

The suitable habitats of the Bharal were mainly distributed in the central part of SNNR, mainly including the southeast part of Yangtze River Park and most of Mekong River Park (Figure 3a). The Tibetan gazelle were suitable for distribution in the central and southwest of SNNR, including the middle and east of Yangtze River Park, the middle and north of Mekong River Park, and most of Yellow River Park (Figure 3b). The Tibetan wild ass was suitable for distribution in the central and eastern regions of SNNR, including the central and eastern regions of Yangtze River Park, the central and northern regions of Mekong River Park, and the most of Yellow River Park (Figure 3c).

In addition, the suitable habitats of the Tibetan antelopes were mainly distributed in the western area of SNNR, including the central and western areas of Yangtze River Park (Figure 3d). The white‐lipped deer were suitable for distribution in the central and southern part of SNNR, including the central and eastern parts of Yangtze River Park, the most of Mekong River Park, and the most of Yellow River Park (Figure 3e). The wild yaks were suitable for distribution in the central and western regions of SNNR, including most areas of Yangtze River Park and the northern area of Mekong River Park (Figure 3f).

In the SNNR and the SNP, the areas and proportions of the six wild herbivores with high, medium, low suitability habitat and unsuitability were shown in Table 3. The results exposed that the proportion of highly suitable and moderately suitable areas for six wild herbivores in the SNP was higher than that in SNNR. The suitable area and proportion of the wild yak and the Tibetan gazelle were higher than other species (Figure 3g,h).

**TABLE 3:** Habitat composition of the six wild herbivores in each park of the Sanjiangyuan National Park (SNP) and Sanjiangyuan National Nature Reserve (SNNR). HSH, MSH, PSH, and USH stand for highly suitable habitat, medium suitable habitat, poorly suitable habitat, and unsuitable area, respectively.

	Species	Area (km^2^)				Percentage (%)			
		HSH	MSH	PSH	USH	HSH	MSH	PSH	USH
SNP	Bharal	8377.08	15,288.44	26,505.11	72,929.37	6.81	12.42	21.53	59.24
	Tibetan gazelle	22,889.18	32,623.99	17,651.84	49,934.99	18.59	26.50	14.34	40.56
	Tibetan wild ass	11,170.38	19,807.65	21,220.08	70,901.90	9.07	16.09	17.24	57.60
	Tibetan antelope	7990.66	16,331.98	29,066.97	69,710.38	6.49	13.27	23.61	56.63
	White‐lipped deer	11,784.02	25,360.58	33,452.93	52,502.47	9.57	20.60	27.18	42.65
	Wild yak	410,58.80	26,171.59	27,940.76	27,928.84	33.35	21.26	22.70	22.69
	Protection level strategy	14,511.40	39,715.58	50,806.71	18,066.31	11.79	32.26	41.27	14.68
	Carrying capacity strategy	18,073.32	43,198.26	44,876.48	16,951.94	14.68	35.09	36.46	13.77
SNNR	Bharal	21,279.69	39,492.57	77,201.02	213,640.53	6.05	11.23	21.96	60.76
	Tibetan gazelle	27,482.02	68,821.83	63,536.09	191,773.85	7.82	19.57	18.07	54.54
	Tibetan wild ass	13,421.57	31,966.87	53,278.81	252,946.55	3.82	9.09	15.15	71.94
	Tibetan antelope	8007.50	17,355.89	42,384.75	283,865.67	2.28	4.94	12.05	80.73
	White‐lipped deer	26,375.37	59,147.33	95,947.59	170,143.51	7.50	16.82	27.29	48.39
	Wild yak	53,396.32	48,312.79	88,779.10	161,125.59	15.19	13.74	25.25	45.82
	Protection level strategy	15,228.83	63,592.20	138,135.97	134,656.80	4.33	18.09	39.29	38.30
	Carrying capacity strategy	19,609.88	81,471.96	124,080.42	126,451.53	5.58	23.17	35.29	35.96

### 3.4 Identification of priority reserves

**FIGURE 4:**
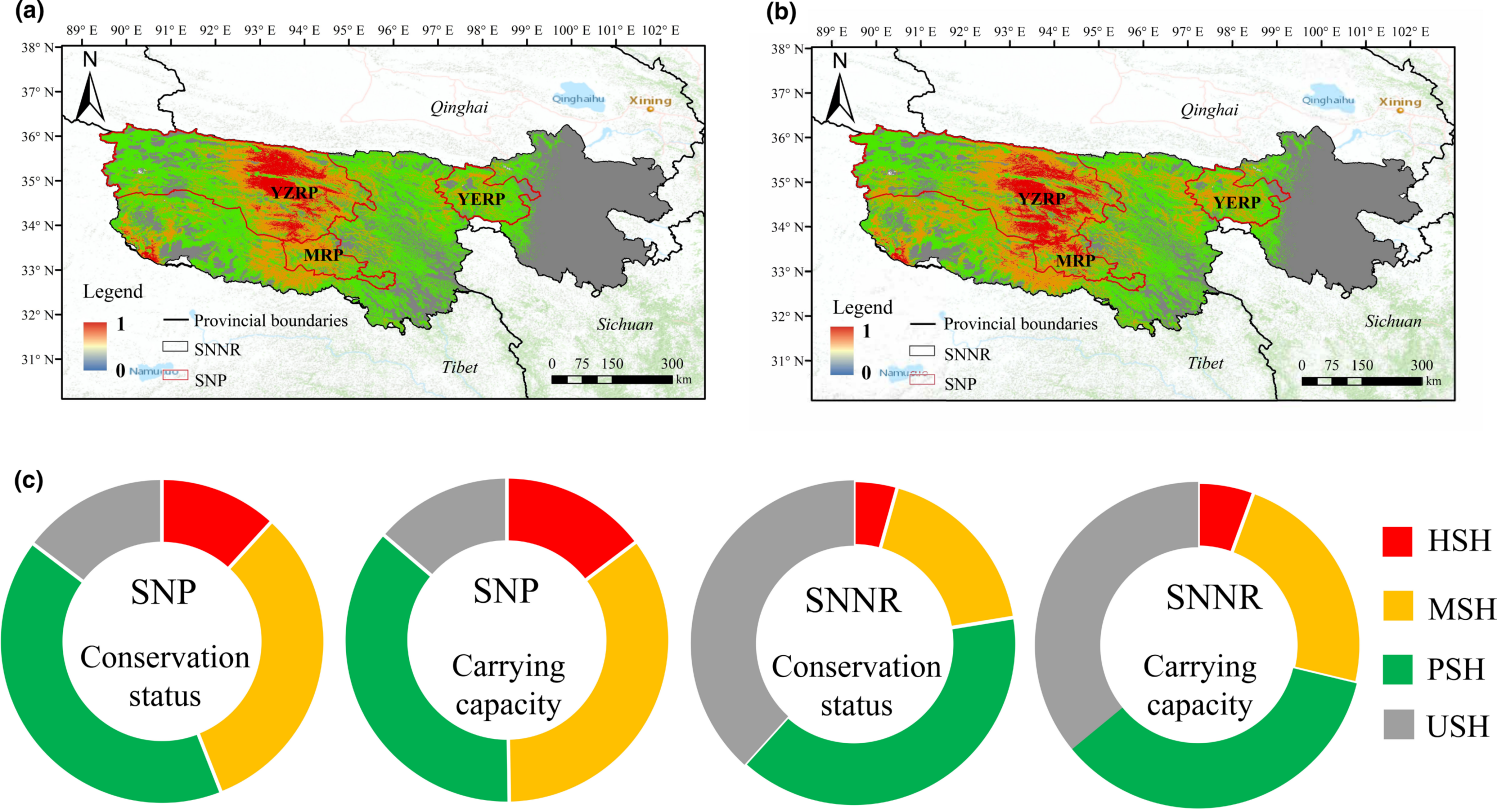
Analysis of priority reserves for the six wild herbivores in the Sanjiangyuan National Park (SNP) (a) and Sanjiangyuan National Nature Reserve (SNNR) (b). (c) Habitat proportion composition after superposition analysis based on different strategies. YZRP, MRP, and YERP stand for Yangtze River park, Mekong River park, and Yellow River Park, respectively.

Based on protection level and carrying capacity strategies, the superposition analysis of suitable habitats of six wild herbivores showed that the distribution pattern of the different strategies was similar (Figure 4). The priority reserves (highly and medium suitable habitat) for wild herbivores were mainly distributed in the middle east, middle west, and southwest of SNNR. The SNP was the main area of the core area. The area around the SNP and between three parks also served as a core reserve for herbivores (Figure 4a,b). Based on protection level strategy, the priority reserves in SNNR covers an area of 78,821.03 km^2^, accounting for 22.42%, of which 54,226.98 km^2^ was located in the SNP (Table 3, Figure 4c). Based on carrying capacity strategy, the priority reserves in SNNR covered an area of 101,081.43 km^2^, accounting for 28.75%, of which 61,271.58 km^2^ was located in the SNP (Table 3, Figure 4c).

The comprehensive analysis indicated that the proportion of highly and medium suitable areas for wild herbivores in the SNP was much higher than that in SNNR.

## 4 DISCUSSION

The study indicates that the average altitude of the six large wild herbivores was above 4300 m, and the optimum elevation range was 4000–5000 m, which was consistent with previous studies. The average altitude of the suitable habitat for the Tibetan antelope and wild yak was relatively higher, while that of the suitable habitat for the Tibetan wild ass was relatively lower (Table S4). Previous studies have shown that the Tibetan antelope is one of gregarious plateau animals living in high altitude areas, suitable for distribution in high altitude alpine grasslands, rolling hills, plateaus, and mountain valleys between 3250 and 5500 m. The suitable habitats for this species are composed of alpine grasslands, alpine desert, and meadows, characterized by low vegetation coverage and low primary productivity (Leslie & Schaller, 2008). Moreover, the wild yak is suitable to be distributed in the alpine desert, plateau meadow, scrub, and other areas in the north of the Qinghai‐Tibet Plateau from 4000 to 6100 m (Buzzard & Berger, 2016). The Tibetan wild ass prefers to live in the alpine grassland, alpine desert grassland, mountain desert, and swampy areas from 2700 to 5400 m (Shah et al., 2015). Since the Bharal is one of the main preys of the top predator of the snow leopard in the alpine ecosystem (Aryal et al., 2014), they usually occur in steep alpine bare rock or alpine meadows at altitudes of 2500–5500 m (Harris, 2014). The Tibetan gazelle generally lives in the alpine grassland, sub‐alpine meadow, and alpine desert with altitude ranging from 3000 to 5750 m (Hu et al., 2015). In addition, the white‐lipped deer prefer to inhabit in alpine semi‐desert steppe, alpine meadow steppe, shrub, mountain forest, open gully, and mountain range with an altitude of 3500–5100 m (Harris, 2015).

Furthermore, this study also indicates that the mean average annual temperature of the distribution areas of six large wild herbivores was below −3.0°C, which was closely related to the suitable habitat at high altitude. The altitude of suitable habitat for the Tibetan antelope and the wild yak was the highest, and correspondingly, the temperature of suitable habitat for these two species was the lowest (Table S4, Figure 2). This study also noted that the average annual precipitation in the range the six herbivores was between 200 and 400 mm, indicating that these species were mainly distributed in semi‐arid regions. Among land use types, grassland and unused land were the highest proportion of these species. Grassland is the largest type of land use in Qinghai province, accounting for more than 50% (Wang et al., 2017), which can provide abundant food resources and shelter for the large wild herbivores in the area. Among vegetation types, they shared a preference for meadow and steppe.

According to the overlapping analysis of habitat suitability of six wild herbivores in the park based on the two strategies of protection level and carrying capacity, the proportion of core protected areas for herbivores in the SNP was much higher than that in the SNNR. This study showed that most of the core areas were located in SNP. The habitat and medium suitable habitats can be used as the core wildlife protection areas of the SNP (Jiang et al., 2019). The core reserve can be obtained by superimposing suitable habitats of multiple species (Wauchope et al., 2017; Whitehead et al., 2014). However, there are also some areas between Yellow River Park, Yangtze River Park, and Mekong River Park, as well as some areas in the southwest of Yangtze River Park, which can also be regarded as core areas. These areas were not included in SNP. At present, the pilot scope of Sanjiangyuan National Park is not connected in space. The headwaters of the Yangtze River and the Yellow River are not fully covered by the national park, not are some important ecosystems and wildlife habitats located at the headwaters. The representativeness, authenticity, and integrity of the ecosystem are not outstanding enough. It is suggested to optimize the functional areas and adjust the boundary scope on the basis of the pilot scope of the Sanjiangyuan National Park system, and integrate it into all headwaters of the Yangtze river, the Yellow River, and the Mekong River, so as to enhance the integrity and connectivity of all functional areas.

SNP is one of the most important biodiversity conservation areas in the world with its unique alpine and alpine climate. It is rich in diverse cultural resources and has formed a typical ecological and cultural system. The park's special geographical location makes it of great and far‐reaching strategic significance in China's national park construction and ecological civilization system construction. The construction of the SNP shoulders the mission of accumulating experience for China's ecological civilization system and providing demonstration for the construction of national parks (Wang, 2019). It has played an irreplaceable role and made important contributions to the establishment of China's natural reserve system with national parks as the main body. Therefore, it is a necessary to carry out functional regionalization and regional optimization research for the scientific construction of national parks. According to the Master Plan of the SNP formulated by the Chinese government in 2018, the boundary and functional zoning of the SNP should be further optimized to ensure the complete system protection of the source of Sanjiangyuan. However, the Yellow River Park was separate from Yangtze River Park and Mekong River Park and was not integrated into one in the Sanjiangyuan area. Some important ecosystems and wildlife habitats in the source areas are not included in the current SNP and are not under the strictest protection. Our study showed many areas outside SNP were also suitable distribution areas for the distribution of the Bharal, the Tibetan gazelle, the Tibetan wild ass, white‐lipped deer, and wild yaks (Figure 2). There is a certain gap between the national park and the original integrity of the protection requirements, its national representative, the integrity of the original is not outstanding.

Wildlife protection research is a hot research field in conservation biology (Gong et al., 2020). Habitat protection is as important as wildlife protection, and habitat assessment is an important part of wildlife protection and biodiversity conservation (Whitehead et al., 2014). The incorporation of wildlife habitat planning into the core protected areas of national parks is an indispensable concrete measure to reflect the authenticity and integrity of national parks. The SNP is an important habitat for endemic species on the plateau. In accordance with the principle of strict protection of the ecosystem, the areas requiring more strict protection are classified into the core protection area, including the important habitat for wildlife at source. It focuses on the strict protection of habitat integrity, as far as possible to avoid regional fragmentation. In recent years, our research team has organized a large number of background surveys of wildlife and further grasped the distribution, quantity, suitable habitat, and other basic information of wildlife in the SNP. Based on the distribution of important wildlife habitats, the spatial suitability analysis of the wildlife in the Sanjiangyuan can provide the most direct reference for the division, functional zoning, and boundary optimization of the core reserves of the wildlife protection in the SNP. On the basis of the existing scope of the SNP, it is the basic requirement of implementing more strict ecological protection and ensuring the integrity of the SNP to optimize and adjust and fully include the overlapping areas suitable for various kinds of wildlife in each park.

## AUTHOR CONTRIBUTIONS

**Feng Jiang:** Conceptualization (equal); data curation (equal); investigation (equal); methodology (equal); resources (equal); software (equal); writing – original draft (equal); writing – review and editing (equal). **Jingjie Zhang:** Conceptualization (equal); data curation (equal); formal analysis (equal); investigation (equal); methodology (equal); software (equal); supervision (equal); visualization (equal); writing – original draft (equal). **Pengfei Song:** Data curation (equal); investigation (equal); methodology (equal); software (equal); validation (equal); visualization (equal). **Wen Qin:** Formal analysis (equal); investigation (equal); resources (equal); software (equal). **Haijing Wang:** Formal analysis (equal); investigation (equal); software (equal). **Zhenyuan Cai:** Data curation (equal); formal analysis (equal); funding acquisition (equal). **Hongmei Gao:** Data curation (equal); investigation (equal); methodology (equal). **Daoxin Liu:** Data curation (equal); investigation (equal); resources (equal). **Bin Li:** Data curation (equal); investigation (equal). **Tongzuo Zhang:** Conceptualization (equal); funding acquisition (lead); investigation (equal); project administration (lead); resources (equal); supervision (equal); validation (equal); writing – original draft (equal); writing – review and editing (equal).

## CONFLICT OF INTEREST

The authors declare that they have no competing interests.

## Supporting information


Appendix S1

## Data Availability

The environment variables used in the study were obtained from WorldClim (http://www.worldclim.com/), Resource and environment data cloud platform (http://www.resdc.cn/), Socioeconomic Data and Applications Center (https://sedac.ciesin.columbia.edu/). Data sharing is not applicable to this article as no new data were created or analyzed in this study.
