# Supplementary material for: Identifying priority reserves favors the sustainable development of wild ungulates and the construction of Sanjiangyuan National Park
Source: Ecol Evol. 2022 Nov 3;12(11):e9464. doi: 10.1002/ece3.9464 (PMC9631328; doi:10.1002/ece3.9464)
Supplement: Supplementary file 1 — Appendix S1 [file ECE3-12-e9464-s001.docx]

**Table S1** Four types of environmental variables used for ecological niche modeling of six large wild herbivorous.

| Type of variable | Variables | Code | Source |
| --- | --- | --- | --- |
| Climate variables | Annual mean temperature | Bio1 | WorldClim (<http://www.worldclim.com/>) |
|  | Mean diurnal range | Bio2 | WorldClim (<http://www.worldclim.com/>) |
|  | Isothermality (Bio2/Bio7) (*100) | Bio3 | WorldClim (<http://www.worldclim.com/>) |
|  | Temperature seasonality (SD*100) | Bio4 | WorldClim (<http://www.worldclim.com/>) |
|  | Max temperature of warmest month | Bio5 | WorldClim (<http://www.worldclim.com/>) |
|  | Min temperature of coldest month | Bio6 | WorldClim (<http://www.worldclim.com/>) |
|  | Temperature annual range (Bio5-Bio6) | Bio7 | WorldClim (<http://www.worldclim.com/>) |
|  | Mean temperature of wettest quarter | Bio8 | WorldClim (<http://www.worldclim.com/>) |
|  | Mean temperature of driest quarter | Bio9 | WorldClim (<http://www.worldclim.com/>) |
|  | Mean temperature of warmest quarter | Bio10 | WorldClim (<http://www.worldclim.com/>) |
|  | Mean temperature of coldest quarter | Bio11 | WorldClim (<http://www.worldclim.com/>) |
|  | Annual precipitation | Bio12 | WorldClim (<http://www.worldclim.com/>) |
|  | Precipitation of wettest month | Bio13 | WorldClim (<http://www.worldclim.com/>) |
|  | Precipitation of driest month | Bio14 | WorldClim (<http://www.worldclim.com/>) |
|  | Precipitation seasonality (*CV*) | Bio15 | WorldClim (<http://www.worldclim.com/>) |
|  | Precipitation of wettest quarter | Bio16 | WorldClim (<http://www.worldclim.com/>) |
|  | Precipitation of driest quarter | Bio17 | WorldClim (<http://www.worldclim.com/>) |
|  | Precipitation of warmest quarter | Bio18 | WorldClim (<http://www.worldclim.com/>) |
|  | Precipitation of coldest quarter | Bio19 | WorldClim (<http://www.worldclim.com/>) |
|  | Minimum temperature from January to December | Tmin1-12 | WorldClim (<http://www.worldclim.com/>) |
|  | Maximum temperature from January to December | Tmax1-12 | WorldClim (<http://www.worldclim.com/>) |
|  | Mean temperature from January to December | Tmean1-12 | WorldClim (<http://www.worldclim.com/>) |
|  | Precipitation from January to December | Prec1-12 | WorldClim (<http://www.worldclim.com/>) |
| Terrain variables | Altitude | Alt | Extraction based on digital elevation model (DEM) data. Resource and environment data cloud platform (<http://www.resdc.cn/>) |
|  | Slop | Slop | Extraction based on DEM |
|  | Aspect | Asp | Extraction based on DEM |
|  | Ruggedness | TRI | Extraction based on DEM |
|  | Curvature | Cur | Extraction based on DEM |
|  | Flow direction | FD | Extraction based on DEM |
| Vegetation variables | Vegetation | Veg | Resource and environment data cloud platform (<http://www.resdc.cn/>) |
|  | Normalized difference vegetation index | NDVI | Resource and environment data cloud platform (<http://www.resdc.cn/>) |
| Human interference | Human influence index | HII | Socioeconomic Data and Applications Center  (<https://sedac.ciesin.columbia.edu/>) |
|  | Population | Pop | Resource and environment data cloud platform (<http://www.resdc.cn/>) |

Figure S1 ROC curves for accuracy of six large wild herbivores prediction using MaxEnt. (a) Bharal; (b) Tibetan gazelle; (c) Tibetan wild ass; (d) Tibetan antelope; (e) White-lipped deer; (f) Wild yak.


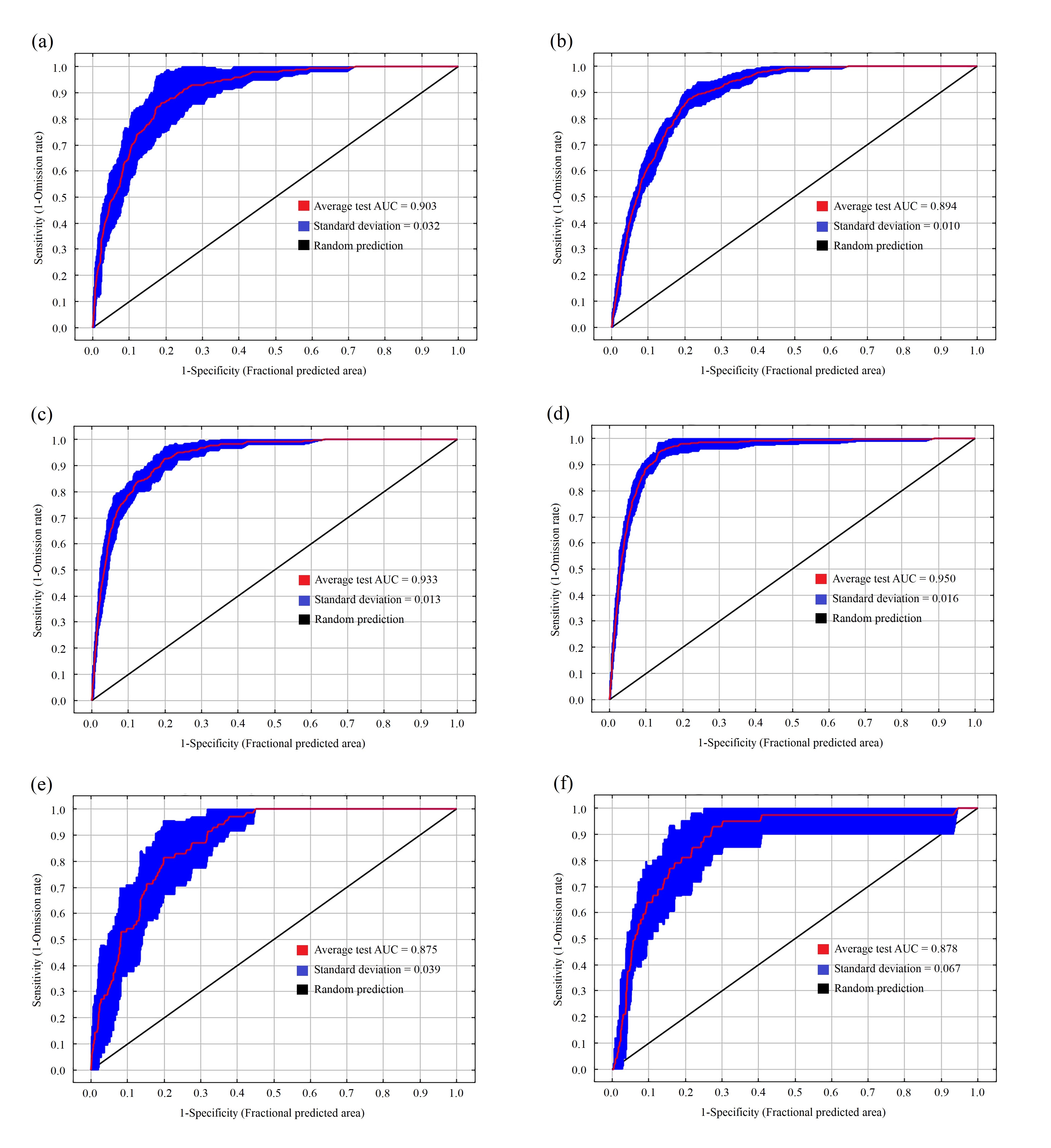


**Table S2** Environmental variables with small correlation coefficients for six large wild herbivores.

IEV stands for initial environmental variables. Variables are derived from terrain (*TE*), the temperatures (*T*), precipitation (*P*), human interference (*HI*), land type (*LT*) or vegetation types (VT). BH, TG, TWA, TA, WLD and WY stands for the Bharal, the Tibetan gazelle, the Tibetan wild ass, the Tibetan antelope, the white-lipped deer and wild yak, respectively. Y indicates that the variable was selected, and N indicates that the variable was not selected.

| Environment variables | *IEV* | BH | TG | TWA | TA | WLD | WY |
| --- | --- | --- | --- | --- | --- | --- | --- |
| Altitude | *TE* | Y | Y | Y | Y | Y | Y |
| Annual Mean Temperature （Bio1）*^a^* | *T* | Y | N | Y | N | Y | N |
| Mean Diurnal Range（Bio2）*^b^* | *T* | Y | Y | Y | Y | Y | Y |
| Isothermality（Bio3）*^b^* | *T* | Y | Y | Y | Y | Y | Y |
| Temperature Seasonality（Bio4）*^a^* | *T* | N | Y | Y | N | N | Y |
| Min Temperature of Coldest Month（Bio6） | *T* | N | Y | N | N | N | Y |
| Temperature Annual Range （Bio7）*^b^* | *T* | Y | N | N | Y | N | N |
| Mean Temperature of Coldest Quarter（Bio11）*^c^* | *T* | N | N | N | N | N | Y |
| Annual Precipitation（Bio12）*^d^* | *P* | N | Y | Y | Y | Y | N |
| Precipitation of Driest Month（Bio14） | *P* | Y | Y | N | Y | N | N |
| Precipitation Seasonality （Bio15）*^d^* | *P* | Y | N | N | Y | Y | Y |
| Precipitation of Driest Quarter（Bio17）*^e^* | *P* | N | N | N | N | N | Y |
| December maximum temperature （Tmax12） | *T* | N | N | Y | N | N | N |
| January precipitation（Prec1） | *P* | N | N | N | Y | N | N |
| March precipitation（Prec3） | *P* | Y | N | N | Y | N | N |
| April precipitation（Prec4） | *P* | N | N | Y | N | N | N |
| Slop | *TE* | Y | Y | Y | Y | Y | Y |
| Aspect | *TE* | Y | Y | Y | Y | Y | Y |
| Curvature | *TE* | Y | Y | Y | Y | Y | Y |
| Flow direction | *TE* | Y | Y | Y | Y | Y | Y |
| Human Influence Index (HII) | *HI* | Y | Y | Y | Y | Y | Y |
| Land cover | *LT* | Y | Y | Y | Y | Y | Y |
| Normalized difference vegetation index (NDVI) | *VT* | Y | Y | Y | Y | Y | N |
| Population | *HI* | Y | Y | Y | Y | Y | Y |
| Vegetation | *VT* | Y | Y | Y | Y | Y | Y |

*^a^* Bioclimatic variable reflects the average temperature and its range.

*^b^* Bioclimatic variable reflects the temperature difference characteristics.

*^c^* Bioclimatic variable reflects the effects of extreme temperatures.

*^d^* Bioclimatic variable reflects the precipitation and its seasonality.

*^e^* Bioclimatic variable reflects the characteristics of hydrothermal synchronization.

**Table S3** Optimal model parameter selection of six large wild herbivores and the specificity of the models. L, Q and P stand for linear, quadratic and product features, respectively. β means the regularization multipliers. AUC_test_ is the average test AUC and SD10 is the standard deviation for 10 repetitions runs.

| Species | Feature class | β | AUC_test_ | SD_10_ |
| --- | --- | --- | --- | --- |
| Bharal | L, Q, P | β=4.0 | 0.903 | 0.032 |
| Tibetan gazelle | L, Q, P | β=2.5 | 0.894 | 0.010 |
| Tibetan wild ass | L, Q, P | β=3.0 | 0.933 | 0.013 |
| Tibetan antelope | L, Q, P | β=3.0 | 0.950 | 0.016 |
| White-lipped deer | L, Q | β=1.0 | 0.875 | 0.039 |
| Wild yak | L, Q | β=3.0 | 0.878 | 0.067 |

**Table S4** Statistical analysis of altitude, annual mean temperature and annual precipitation for six wild herbivores in Sanjiangyuan area. Min., Max. and SD stand for minimum, maximum and standard deviation, respectively. *CV* stand for the coefficient of variation.

| Species | Environmental variables | Min. | Max. | Mean | SD | *CV* (%) | Optimum range |
| --- | --- | --- | --- | --- | --- | --- | --- |
| Bharal | Altitude (Alt; m) | 3434 | 5117 | 4561.3 | 269.0 | 5.9 | 4400-4900 (70.2%) |
|  | Annual Mean Temperature (Bio1; ℃) | -7.2 | 2.5 | -3.1 | 1.9 | -59.5 | [-5, -1) (75.1%) |
|  | Annual precipitation (Bio12; mm) | 94 | 538 | 397.2 | 95.6 | 24.1 | 300-550 (85.4%) |
| Tibetan gazelle | Altitude (Alt; m) | 3552 | 5093 | 4458.8 | 240.5 | 5.4 | 4200-4700 (74.2%) |
|  | Annual Mean Temperature (Bio1; ℃) | -7.2 | 1.9 | -3.6 | 1.2 | -34.0 | [-5, -2) (84.1%) |
|  | Annual precipitation (Bio12; mm) | 105 | 529 | 320.8 | 75.1 | 23.4 | 200-400 (81.1%) |
| Tibetan wild ass | Altitude (Alt; m) | 3739 | 4958 | 4352.6 | 199.0 | 4.6 | 4100-4600 (82.9%) |
|  | Annual Mean Temperature (Bio1; ℃) | -6.7 | 0.2 | -3.2 | 0.8 | -24.0 | [-5, -2) (95.0%) |
|  | Annual precipitation (Bio12; mm) | 116 | 523 | 341.4 | 48.3 | 14.1 | 300-400 (83.2%) |
| Tibetan antelope | Altitude (Alt; m) | 2686 | 5130 | 4640.3 | 271.4 | 5.8 | 4400-5000 (96.4%) |
|  | Annual Mean Temperature (Bio1; ℃) | -7.6 | 4.7 | -5.0 | 1.4 | -28.2 | [-7, -3) (97.4%) |
|  | Annual precipitation (Bio12; mm) | 19 | 384 | 226.5 | 38.5 | 17.0 | 150-300 (97.9%) |
| White-lipped deer | Altitude (Alt; m) | 3569 | 4968 | 4511.6 | 285.5 | 6.3 | 4300-4900 (78.6%) |
|  | Annual Mean Temperature (Bio1; ℃) | -6.2 | 2.2 | -3.0 | 1.6 | -53.8 | [-5, -1) (82.9%) |
|  | Annual precipitation (Bio12; mm) | 113 | 534 | 380.2 | 107.1 | 28.2 | 250-500 (87.1%) |
| Wild yak | Altitude (Alt; m) | 3869 | 5000 | 4640.0 | 245.4 | 5.3 | 4500-5000 (83.7%) |
|  | Annual Mean Temperature (Bio1; ℃) | -7.3 | 1.3 | -4.8 | 1.8 | -37.0 | [-7, -2) (91.8%) |
|  | Annual precipitation (Bio12; mm) | 127 | 532 | 247.9 | 98.2 | 39.6 | 150-250 (65.3%) |

**Table S5** Correlation analysis of altitude of six large wild herbivores. **P* < 0.05 (Pearson), ***P* < 0.01.

| Species | BH | TG | TWA | TA | WLD | WY | SNP | SNNR |
| --- | --- | --- | --- | --- | --- | --- | --- | --- |
| Bharal (BH) | 1 |  |  |  |  |  |  |  |
| Tibetan gazelle (TG) | 0.726** | 1 |  |  |  |  |  |  |
| Tibetan wild ass (TWA) | 0.313 | 0.788** | 1 |  |  |  |  |  |
| Tibetan antelope (TA) | 0.794** | 0.642** | 0.132 | 1 |  |  |  |  |
| White-lipped deer (WLD) | 0.915** | 0.784** | 0.318 | 0.838** | 1 |  |  |  |
| Wild yak (WY) | 0.575** | 0.554* | 0.066 | 0.837** | 0.722** | 1 |  |  |
| Sanjiangyuan National Park (SNP) | 0.849** | 0.588** | 0.204 | 0.843** | 0.810** | 0.666** | 1 |  |
| Sanjiangyuan National Nature Reserve (SNNR) | 0.906** | 0.702** | 0.312 | 0.830** | 0.878** | 0.663** | 0.971** | 1 |

**Table S6** Correlation analysis of annual mean temperature of six large wild herbivores. **P* < 0.05 (Pearson), ***P* < 0.01.

| Species | BH | TG | TWA | TA | WLD | WY | SNP | SNNR |
| --- | --- | --- | --- | --- | --- | --- | --- | --- |
| Bharal (BH) | 1 |  |  |  |  |  |  |  |
| Tibetan gazelle (TG) | 0.795** | 1 |  |  |  |  |  |  |
| Tibetan wild ass (TWA) | 0.587* | 0.945** | 1 |  |  |  |  |  |
| Tibetan antelope (TA) | 0.651* | 0.39 | 0.102 | 1 |  |  |  |  |
| White-lipped deer (WLD) | 0.900** | 0.943** | 0.844** | 0.405 | 1 |  |  |  |
| Wild yak (WY) | 0.603* | 0.499 | 0.301 | 0.868** | 0.492 | 1 |  |  |
| Sanjiangyuan National Park (SNP) | 0.672* | 0.699** | 0.502 | 0.813** | 0.623* | 0.728** | 1 |  |
| Sanjiangyuan National Nature Reserve (SNNR) | 0.861** | 0.828** | 0.635* | 0.736** | 0.834** | 0.673* | 0.922** | 1 |

**Table S7** Correlation analysis of annual precipitation of six large wild herbivores. **P* < 0.05 (Pearson), ***P* < 0.01.

| Species | BH | TG | TWA | TA | WLD | WY | SNP | SNNR |
| --- | --- | --- | --- | --- | --- | --- | --- | --- |
| Bharal (BH) | 1 |  |  |  |  |  |  |  |
| Tibetan gazelle (TG) | 0.533* | 1 |  |  |  |  |  |  |
| Tibetan wild ass (TWA) | 0.499 | 0.879** | 1 |  |  |  |  |  |
| Tibetan antelope (TA) | -0.17 | 0.218 | -0.114 | 1 |  |  |  |  |
| White-lipped deer (WLD) | 0.841** | 0.481 | 0.305 | -0.083 | 1 |  |  |  |
| Wild yak (WY) | -0.153 | 0.159 | -0.078 | 0.843** | -0.168 | 1 |  |  |
| Sanjiangyuan National Park (SNP) | 0.286 | 0.709** | 0.452 | 0.690** | 0.242 | 0.749** | 1 |  |
| Sanjiangyuan National Nature Reserve (SNNR) | 0.752** | 0.736** | 0.526* | 0.362 | 0.632* | 0.358 | 0.736** | 1 |

**Table S8** Correlation analysis of land use types of six large wild herbivores. **P* < 0.05 (Spearman), ***P* < 0.01.

| Species | BH | TG | TWA | TA | WLD | WY | SNP | SNNR |
| --- | --- | --- | --- | --- | --- | --- | --- | --- |
| Bharal (BH) | 1 |  |  |  |  |  |  |  |
| Tibetan gazelle (TG) | 0.832* | 1 |  |  |  |  |  |  |
| Tibetan wild ass (TWA) | 0.941** | 0.955** | 1 |  |  |  |  |  |
| Tibetan antelope (TA) | 0.882* | 0.893* | 0.941** | 1 |  |  |  |  |
| White-lipped deer (WLD) | 0.985** | 0.907* | 0.985** | 0.925** | 1 |  |  |  |
| Wild yak (WY) | 0.882* | 0.893* | 0.941** | 1.000** | 0.925** | 1 |  |  |
| Sanjiangyuan National Park (SNP) | 0.928** | 0.941** | 0.986** | 0.928** | 0.971** | 0.928** | 1 |  |
| Sanjiangyuan National Nature Reserve (SNNR) | 0.928** | 0.941** | 0.986** | 0.928** | 0.971** | 0.928** | 0.943** | 1 |

**Table S9** Correlation analysis of vegetation types of six large wild herbivores. **P* < 0.05 (Spearman), ***P* < 0.01.

| Species | BH | TG | TWA | TA | WLD | WY | SNP | SNNR |
| --- | --- | --- | --- | --- | --- | --- | --- | --- |
| Bharal (BH) | 1 |  |  |  |  |  |  |  |
| Tibetan gazelle (TG) | 0.623 | 1 |  |  |  |  |  |  |
| Tibetan wild ass (TWA) | 0.843** | 0.921** | 1 |  |  |  |  |  |
| Tibetan antelope (TA) | 0.393 | 0.951** | 0.801* | 1 |  |  |  |  |
| White-lipped deer (WLD) | 0.898** | 0.645 | 0.849** | 0.513 | 1 |  |  |  |
| Wild yak (WY) | 0.327 | 0.48 | 0.483 | 0.563 | 0.686 | 1 |  |  |
| Sanjiangyuan National Park (SNP) | 0.714* | 0.970** | 0.952** | 0.896** | 0.743* | 0.518 | 1 |  |
| Sanjiangyuan National Nature Reserve (SNNR) | 0.810* | 0.862** | 0.940** | 0.761* | 0.874** | 0.6 | 0.952** | 1 |
